# On Infantile Convulsions Arising from Spasm of the Intestines

**Published:** 1829-05

**Authors:** Jos. Parrish

**Affiliations:** one of the Surgeons of the Pennsylvania Hospital, &c.


### INFANTILE CONVULSIONS.

*On Infantile Convulsions arising from Spasm of the Intestines.* By Jos. Parrish, m.d. one of the Surgeons of the Pennsylvania Hospital, &c.

Among the disagreeable effects produced by intestinal irritation in infants, is a species of convulsion resembling the epileptic fit in appearance, but materially differing from the ordinary form of that disease, both in its origin and the mode of treatment which it demands. A character by which it may, in most instances, be readily distinguished from genuine epilepsy, is the total absence of stupor, after the cessation of the convulsions. Instead of remaining for some time in a comatose state, the child, upon recovering from the fit, immediately becomes entirely sensible, and looks as though nothing had happened. The attacks are generally sudden and of short continuance, and in the beginning are seldom frequent. Sometimes two or more will take place in quick succession; and days, and even weeks, will elapse before the return of the convulsions. If not arrested, however, they become more frequent; and at length the nervous system is rendered so excessively irritable, that an almost constant convulsive action is kept up, till the tender frame of the infant becomes exhausted, and sinks under the disease. So far, however, as I have seen, the affection generally yields to proper treatment; and a relapse may be prevented by attention to the diet and the state of the bowels. The infant is usually attacked within the year; and, if it survive the period of dentition, may be considered safe.

That the primary affection is seated in the bowels, and consists of spasm of its muscular coat, is evinced by the symptoms which are presented in the intervals of the convulsions. The child often screams out suddenly, throws itself back, and stiffens its body, exactly as in an attack of flatulent colic; and the comparative ease which it regains in a few minutes proves the affection to have been spasmodic. Even when the pain is less severe, the peculiar motions and complaints of the infant are such as experienced mothers and nurses immediately attribute to uneasiness in the cavity of the abdomen. The relief which sometimes follows discharges of flatus is another proof, if more were wanting, of the nature of the complaint. Indeed, the extrication of air in the bowels is often very abundant; so much so that the abdomen swells, and even becomes tympanitic. The nature of the discharges also indicates disorder in the alimentary canal; and the existence of acidity is frequently manifested, as well by the sourness of the breath as by the smell and appearance of the stools. It is of the utmost importance to observe accurately these symptoms of abdominal affection, as they both enable us to form a correct view of the nature of the convulsions, and at the same time point out the course of treatment proper for their removal.

In a case which fell under my care in the year 1821, and which unfortunately proved fatal, I had an opportunity of verifying my views of the nature of the disease by dissection. The subject of the attack was an infant about five months old. The convulsions came on instantaneously, without the least warning; and, immediately after they had passed, the patient was quite sensible, and sometimes even playful. At first, several days intervened between the fits; and, contrary to the general rule, they came on at one stated time, at or a little after daybreak. They afterwards became more frequent and distressing; and, towards the close of the case, severe spasms or partial convulsions occurred in the intervals, the child screaming out, and appearing to be in much pain. The treatment was principally directed to the bowels; but leeches were twice applied to the head, and blisters were placed behind the ears. Though relief was occasionally obtained, yet no permanent impression was produced upon the disease; and, notwithstanding all my efforts, the little sufferer expired. Upon dissection, the bowels exhibited strong evidence of having been under the influence of severe spasm. More than half of the small intestines were irregularly contracted. In some places, for more than an inch in extent, the bowel was reduced to the size of a goosequill; in others it appeared as if tied by a thread, its caliber being almost obliterated. The omentum was closely folded up, in the form of a thick twine or small rope, and lay on the arch of the colon. In the gall-bladder was a light-coloured and glairy fluid. No other sign of disease was visible in the cavity of the abdomen or in the thorax. The condition of the brain I did not examine, as I thought sufficient evidence of the cause of death was afforded by the appearance of the bowels.

Though, from the symptoms during life, and the phenomena presented by dissection, I am inclined to attribute these convulsions to intestinal spasm, yet there can be no doubt that difficult dentition greatly aggravates the disorder. This it does, in all probability, more by its influence upon the bowels, than by its direct operation upon the brain and nerves. Inflammation of the gums in infants is exceedingly apt to produce irritation in the alimentary canal, and, by giving rise to spasm in this part, or aggravating it if already existing, may either serve as a remote cause of the convulsions, or may render them more severe and more difficult to cure. I do not, however, deny that dentition, like any other source of irritation, may produce convulsions by directly operating upon the cerebral system. We undoubtedly meet with instances of the kind; but the symptoms in these cases are materially different from those of the disease under consideration. The attacks are less frequent in their occurrence, are accompanied with greater arterial excitement, and generally exhibit more obvious symptoms of disordered action in the brain.

In the treatment of convulsions from intestinal spasm, we are presented with two obvious indications: namely, to relax the spasm, and to remove those sources of irritation from which it arises. I shall proceed to detail the remedies which I have found most effectual for both purposes. Of those calculated to relax spasm, bloodletting is one of the most important. Though not called for by the condition of the pulse, nor by the existence of any inflammatory action, it sometimes affords great relief, and is useful also by preparing the system for the administration of the antispasmodics. General bleeding is preferable to leeching, as it produces a more prompt and decided impression; but the local abstraction of blood may be resorted to with advantage when the constitution of the child is delicate, and there is reason to apprehend excessive debility from the use of the lancet.

Another important relaxing remedy is the warm bath. This may be used advantageously during the fit, when its duration is more than usually protracted, or the convulsions are uncommonly severe.

But the means upon which I place most reliance are the antispasmodic medicines. Of these I prefer the assafœtida, given very freely, in the shape of emulsion, both internally and by the rectum. In either way it may often be advantageously combined with a little laudanum; especially when, in the intervals of the convulsions, the infant screams much and appears to be in pain. According to the age of the child, from two to five grains of the gum resin may be given every two hours; and, when laudanum is advisable, one or two drops may be added to each dose. In the form of enema, from ten to twenty grains should be administered at once, and repeated more or less frequently, according to circumstances. Sometimes the assafœtida cannot be retained on the stomach, and its very frequent use in the way of injection may produce unpleasant irritation of the rectum. Under these circumstances, the rectified oil of amber may be usefully employed as a substitute; and I have sometimes derived advantage from alternating this medicine with the assafœtida. I prescribe it in the dose of from two to five drops, rubbed up with gum arabic, loaf sugar, and cinnamon water.

Should the case resist the influence of these antispasmodics, or should they lose their effect upon repetition, the musk jalap may be resorted to. In more than one very severe case I have employed it with the most manifest advantage. The proper dose is from half a grain to one grain every hour or two hours, during the greatest violence of the symptoms. If there should be much pain, laudanum should be combined with the oil of amber or musk, as before recommended with the assafœtida. Sometimes more effectual relief will be afforded by the laudanum in the form of anodyne injection, especially when the stomach is irritable. From four to eight drops may be given in this way, and repeated when called for by the symptoms.

At the same time that these remedies are administered internally for the relaxation of the spasm, the use of external measures for the same purpose should not be neglected. In most of the spasmodic complaints of infants, I recommend the application, along the spine, and over the parts more especially affected, of a liniment composed of oil of amber and laudanum, diluted with equal parts of olive oil and brandy.* In the complaint under consideration, decided advantage may be expected from freely bathing the back and abdomen with this liniment. Garlic and brandy, employed in the same way, is also a useful application. In severe cases, the abdomen should be covered with a large blister.

But while we are thus endeavouring to overcome the spasmodic action of the intestines, we should not overlook those sources of irritation, which, if they do not give rise to it, at least render it more severe and obstinate. In the very commencement of the treatment, the contents of the intestines should be freely evacuated; and, throughout the case, accumulations of feculent matters or acrid secretions should

* The proportions which I generally employ are, of the oil of amber and laudanum, each a teaspoonful; of the olive oil and brandy, each a tablespoonful.

be prevented, by the use of castor-oil, magnesia, or some other gentle cathartic.

As in almost every instance there is more or less acid in the stomach and bowels, the frequent administration of alkaline medicines will be found highly useful. I am much in the habit of prescribing the alkaline infusion, prepared, according to the formula of Dr. Physick, from hickory ashes and soot. When symptoms of acidity present themselves, this preparation, diluted so as to suit the palate of the infant, may be given in the dose of a teaspoonful every two or three hours, in alternation with the antispasmodic medicine which may be in use at the time.

The accumulation of flatus has been mentioned as a troublesome attendant of the complaint. This symptom is often greatly relieved by the antispasmodics before recommended; but sometimes it resists all internal remedies, and the intestines become so much distended with air as to be rendered almost incapable of their usual peristaltic action. The removal of so powerful a cause of irritation is absolutely necessary for the preservation of life. For this purpose I generally employ the dry syringe, by which the air may sometimes be readily abstracted, and the abdomen reduced to its natural size. In a case which I attended in consultation with Dr. Wood, and in which the infant was brought to the lowest possible condition short of dissolution, the happiest effects resulted from the use of this instrument. The tympanitic state of the bowels was immediately relieved, opportunity for the continued action of the proper remedies was thus afforded, and the life of the child preserved.*

Another object of attention, so obvious as hardly to deserve particular notice, is the condition of the gums. These, if inflamed, should be freely lanced, and the operation repeated whenever the wounds heal, so long as the continuance of the inflammation may render it advisable. As recommended in a former communication on the subject of cholera, blisters should be applied behind the ears, and kept open by stimulating dressings. In one instance of dangerous convulsions, in which the intestinal disorder appeared to be aggravated, if not altogether maintained, by

* Sometimes the simple introduction of the point of the tube up the rectum answers the purpose; the air escaping by its own elasticity. Sometimes it is necessary to exhaust the air by repeatedly withdrawing the piston of the syringe, and thus creating a vacuum in the body of the instrument, which can be supplied only by the flatus from the bowels. Relief, however, cannot always be obtained in this way.

severe and obstinate inflammation of the gums, great relief was obtained by the application of leeches to the cheeks. The soreness and tumefaction of the gums, which had been excessive, abated almost immediately; the affection of the bowels gave way before the remedies which it had previously resisted; and the convulsions disappeared with the cause upon which they depended.

I will close this account of the treatment by a few observations on the subject of diet. It is manifestly necessary that all articles of food which have a tendency to produce flatulence, or otherwise irritate the bowels, should be carefully avoided. The farinaceous articles, and animal food of easy digestion, are suitable; and the breast may be allowed when the nurse or mother is perfectly healthy. Sometimes the constitution of the mother is such that her milk disagrees with the infant; and, when this can be ascertained, or is strongly suspected, it is proper that a healthy wetnurse should be procured as a substitute. In a respectable family of this city, I was successively called to two of the infant children, affected with convulsions from intestinal spasm, of whom the first recovered, but the second died. Having some reason to attribute the complaint to the milk of the mother, I advised that, upon the birth of a third child, a wetnurse should be immediately provided. My advice was attended to, and the next infant entirely escaped the disorder. In all cases, when the child is allowed to take the breast, the mother should be directed to regulate her diet, and to avoid especially all acescent and flatulent food.

In illustration of the preceding statements, and in order to present in one view a connected account of the symptoms and treatment, I will give the details of a very interesting case, which occurred to me in the year 1818.

Case.—The subject of this note was a remarkably fine boy, aged about nine months. Before I saw him he had been affected with several convulsions, which came on suddenly, and were of short continuance. After one of these attacks he was brought to my house. Finding his gums inflamed and his bowels constipated, I lanced the former, and directed the latter to be freely opened. I also directed the Lac assafœtidæ to be given internally, and the spine to be bathed with garlic and brandy.

Shortly afterwards I was called to see him in the night, in consequence of his having two violent convulsions in pretty quick succession. I found him with a cool skin, and a blue appearance about the mouth, but evidently with a clear intellect. Almost immediately upon coming out of a fit, he appeared to know those to whom he had been accustomed. As his pulse was somewhat tense, I bled him freely; so much so, indeed, that he fainted under the operation. Soon after his revival he was attacked with another fit, which, on his being put into the warm bath, subsided in two minutes. Upon administering some Lac assafœtidæ, I observed the escape of flatus from the stomach; and that the child suffered great pain was manifested by his stiffening himself, and screaming very frequently. I therefore gave him an enema of assafœtida, with nine drops of laudanum; and, upon leaving the house, directed that, if this should not afford relief, one drop of laudanum, with a little assafœtida, should be administered every two hours, till he should become easy. I also recommended that a large spice plaster should be placed upon the abdomen.

The subsequent part of the night was passed more comfortably; but about daybreak he had another convulsion, which was said to be more violent than any of the preceding. He was again put into the warm bath; and a medical friend, who remained with him during the night, gave him a mixture consisting of one drop of laudanum, four drops of the spirits of hartshorn, and a little magnesia, and applied a pair of blisters to his legs.

In the morning I again saw him, and, being impressed with the conviction that much of the mischief depended upon acid in the stomach and bowels, I directed a teaspoonful of the alkaline infusion to be given every two hours, and the mixture above mentioned to be repeated at the same interval, if the child should be in pain. Under this treatment, united with the external application of oil of amber along the spine, and of laudanum and brandy to the abdomen, he passed the day with more comfort, not having to take the laudanum oftener than two or three times.

On repeating my visit, I was encouraged by finding that his skin was becoming warm and his face somewhat flushed. These symptoms were favorable, as they indicated a determination from the internal organs towards the skin.

On the morning of the following day, I was sent for early. One slight convulsion had occurred about midnight, and, from his restlessness, another was apprehended. I watched him closely, and listened to his language; for infants have a language, and happy is the physician who has studied and understands it. I thought I could gather from his mode of expression that the pain had left his bowels, but that he was very uncomfortable and thirsty, and that a little cold water would afford great relief. I accordingly gave him some. He took it with avidity, appeared to be refreshed, and in a short time sunk into a pleasant sleep, which continued most of the morning. In the afternoon he again became uneasy and fretful, with his skin warm and his face flushed. The cool water was renewed; and, as the air appeared to refresh him, I directed he should be carried out into the yard, where he soon fell asleep. I mention these facts to illustrate the importance of attending to the signs by which infants express their wants. Much suffering, and perhaps some aggravation of the disease, were in this instance avoided by the very simple remedies of fresh air and a little cool water.

During the day his gums were lanced, and he took a dose of castor oil. The night was passed comfortably, and without convulsions. Next day, however, he was repeatedly threatened with them, and I found it necessary to administer some powerful antispasmodic. The musk julap was preferred. A dose of this, containing rather less than a grain of the musk, was several times repeated, with very great success. In addition, the bowels were opened by an injection of oil with fennel-seed tea; a spice plaster was placed over the abdomen, and the liniments before mentioned were freely applied. By these means the threatening symptoms were removed, and convulsions warded off; nor did he afterwards experience an attack.

Throughout the case the breast had been denied him, as the mind of the mother was greatly agitated, and I feared that, on this account, her milk might prove injurious. But as she was now more composed, and had for several days confined herself to a strict animal diet, I allowed her again to suckle the child; nor did any unpleasant effects result.

Two of the other children were about this time attacked with catarrhal fever; and I was exceedingly alarmed at finding my little patient, after having already suffered so much, seized with violent symptoms of the same disease. It came on with a chill, which was succeeded by a hot skin, an active pulse, and extreme difficulty of breathing. I do not know that I ever witnessed a case more alarming and apparently dangerous. Much relief, however, was procured by bleeding and an emetic of ipecacuanha. A purge of calomel was afterwards given, followed by small doses of the sweet spirits of nitre and antimonial wine. By the occasional use of the warm bath, by bathing the abdomen with laudanum and brandy, and by strict attention to the state of the bowels, we endeavoured to counteract any disposition which might exist to a return of the convulsions. Under this treatment the child gradually improved, and in the end completely recovered.

It must be evident from the above details that a proper diagnosis of this disease is of the utmost importance, and that treatment founded upon the idea that the brain is the part principally affected will in all probability eventuate in failure. It is also important to understand that these convulsions may occur in infants predisposed to them, during the continuance of another disease; the usual remedies for which will, by the new state of things, be rendered highly injurious. A case illustrative of this fact occurred to me in the year 1822.

The infant was attacked with croup, for which I prescribed the ordinary remedies; such as bleeding, a blister to the throat, calomel, antimonial wine, seneca, &c. Under this treatment the symptoms of the disease had begun to subside, when the child was suddenly seized with severe convulsions. Evident signs of pain in the abdomen were presented, and the state of the stools indicated the existence of acid in the bowels. I was convinced that the convulsions had their origin in intestinal spasm, and, under this impression, immediately laid aside the internal remedies I had prescribed for the croup. In their place I directed the Lac assafœtida, both by the mouth and rectum, the alkaline infusion, and the warm bath. The back and abdomen were frequently bathed with the liniment of oil of amber, laudanum, &c.; and poultices containing the oil of amber were applied to the feet. A surprising change for the better ensued, and the child happily recovered.

As closely connected with the subject of this paper, it will not be improper to notice another effect of spasm of the bowels, which is liable to be mistaken for disease of the brain. I cannot do this better than by communicating the following brief account of a case which fell under my attention. The child was affected with dysenteric symptoms, and in the progress of the disorder became delirious. By the usual treatment these symptoms were relieved, and I considered my patient convalescent. In a short time, however, a change for the worse took place. The bowel affection returned, accompanied with the most distressing derangement of the mental functions. The child was completely maniacal, showing a disposition to snap at and bite, like a dog, whatever approached it. My attention was very naturally directed to the head; and I concluded that some serious cerebral disease must have occurred, to give rise to such a train of symptoms. But the measures suggested by this view of the nature of the complaint were wholly ineffectual, and the little sufferer expired. Having obtained permission to examine the body, I opened the cavity of the cranium, and was surprised to find scarcely a vestige of morbid action in the brain. Within the abdomen, however, were discovered marks of violent spasmodic disease. The small intestines, through almost their whole extent, were irregularly contracted; in many places being diminished, for an inch or two in length, to the size of a worm, in others appearing as if tightly embraced by a ligature. I have no doubt that in this case the maniacal symptoms were the result of excruciating pain arising from violent spasm in the bowels.

On the whole it may be concluded, that the subject of intestinal spasm, involving, as it does, such dangerous consequences, is of the highest interest and importance; and that it more especially claims our minute attention, as, from the delusive nature of the symptoms sometimes presented, and our inadequate means of information relative to the feelings of the patient, we are liable, without much care, to be led into mistaken judgments, and to adopt inefficient, if not injurious modes of treatment.*

* North American Med. and Surg. Journal.

